# New approach to the mitral valve through the left anterior minithoracotomy for combined valve and coronary surgical procedures

**DOI:** 10.1016/j.xjtc.2023.11.015

**Published:** 2023-12-06

**Authors:** Oleksandr Babliak, Vasyl Lazoryshynets, Volodymyr Demianenko, Dmytro Babliak, Anton Marchenko, Katerina Revenko, Yevhenii Melnyk, Oleksii Stohov

**Affiliations:** a Division of Cardiac Surgery, Diagnostic and Treatment Center for Children and Adults of the Dobrobut Medical Network, Kyiv, Ukraine; b National Academy of Medical Sciences, National Amosov Institute of Cardiovascular Surgery of the National Academy of Medical Sciences of Ukraine, Kyiv, Ukraine

**Keywords:** Mitral valve, CABG, TCRAT, Minimally Invasive, left anterior thoracotomy

## Abstract

**Objective:**

We have developed a new technique for accessing the mitral valve through the left anterior minithoracotomy. This approach has been used in patients requiring both mitral valve surgery and coronary artery bypass grafting.

**Methods:**

From October 2020 to September 2022, we performed 24 concomitant mitral valve procedures and coronary artery bypass grafting through the left anterior minithoracotomy. The average age of the patients was 65.5 years, and the mean left ventricular ejection fraction was 44.5%. Computed tomography angiography was routinely performed preoperatively. The surgical technique included a left anterior minithoracotomy in the fourth intercostal space, peripheral cardiopulmonary bypass, aortic crossclamping using a transthoracic clamp through the additional port in the left second intercostal space, the administration of cold blood cardioplegia, a right atrial transseptal approach to the mitral valve, and special surgical exposure maneuvers. These maneuvers were designed to displace the heart into the left pleural space by pulling the inferior vena cava tape and the ascending aorta tape to the left. Conventional mitral valve surgical techniques were used. The mitral valve repair or replacement was performed after the distal anastomoses to the right and circumflex coronary system were completed. Subsequently, after the mitral valve procedure, coronary anastomosis to the left anterior descending artery was performed.

**Results:**

The mitral valve was effectively visualized, and a planned procedure was successfully completed in all patients. There was no need for conversion to a sternotomy. mitral valve repair was performed in 22 patients (91.7%), and mitral valve replacement was performed in 2 patients (8.3%). Conventional surgical instruments were used in 10 cases (41%), and long-shafted instruments were used in 14 cases (59%). A knot-pusher was required in 9 cases (37.5%). A computed tomography distance from the skin level to the mitral valve posterior annulus of more than 14 cm was identified as a technical difficulty marker, necessitating the use of long-shafted instruments. Concomitant complete revascularization was achieved in all cases. The mean number of distal anastomoses was 2.54 ± 0.7 (1; 4). Total operation time was 341 ± 41 (285; 420) minutes, cardiopulmonary bypass time was 231 ± 38 (172; 316) minutes, and the crossclamp time was 127 ± 23 (80; 169) minutes. Patients had a mean intensive care unit stay of 1.87 ± 0.69 (1; 4) days, and their total hospital stay averaged 6.54 ± 1.86 (4; 10) days. There were no reoperations due to bleeding, no occurrences of strokes, and no other major complications. There were no instances of hospital mortality or mortality within 30 days after the procedures.

**Conclusions:**

Mitral valve repair or replacement through the left anterior thoracotomy and transseptal approach is a valuable and effective technique that can be used for concomitant procedures performed through a single minithoracotomy incision in selected patients.

**Figure:**
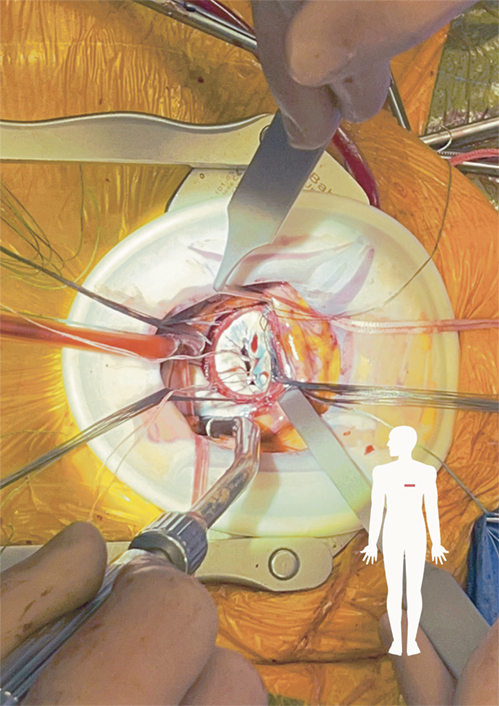
MV exposure through the left anterior thoracotomy in the fourth intercostal space.

**Central Message**

Coronary artery bypass combined with MV repair/replacement can be successfully performed through a single LAmT.

**Perspective**

Performing MV surgery through the left anterior thoracotomy will expand the range of surgical options for patients requiring combined procedures, particularly those who can benefit from a sternum-sparing approach.

See Discussion on page 64.

Traditionally, mitral valve (MV) surgery involved a sternotomy. With advancements in minimally invasive surgical techniques, most isolated MV surgeries can now be safely and effectively performed through a right minithoracotomy approach.^1^ However, procedures that combined MV repair or replacement with coronary artery bypass grafting (CABG) are still typically performed through a median sternotomy. Recently, we have found that the MV can be effectively accessed through the left anterior minithoracotomy (LAmT),^2^ which we routinely use as the primary approach for patients undergoing multivessel CABG.^3^^,^^4^ This discovery has enabled us to perform combined coronary and valve surgical procedures in selective patients through a single minithoracotomy.

The aim of this article is to analyze our novel technique for approaching the MV through the LAmT in patients who require both MV and coronary surgery.

## Materials and Methods

### Study Population

This study represents a retrospective analysis of a cohort of patients treated at a single center. The patients in this cohort underwent minimally invasive MV surgery with access through the right atrium and interatrial septum, along with simultaneous coronary revascularization via LAmT. The procedures were performed between October 2020 and September 2022, using cardiopulmonary bypass (CPB) and cardioplegic cardiac arrest. All patients who underwent procedures with this approach were included in the study. The study cohort comprised 24 patients. Within the same timeframe, 5 patients with the combined MV and coronary procedures underwent sternotomy and were excluded from the study.

According to the Carpentier classification^5^ type I MV insufficiency was the most common, observed in 14 patients (58.3%), followed by type II in 2 patients (8.3%), with no classes of type III A, and type III B in 8 patients (33.3%).

Of the 24 patients, 2 were preoperatively considered for MV replacement using a biological prosthesis, and MV repair techniques were used in the remaining patients. Additionally, tricuspid valve repairs were performed in 3 of these patients. The data were retrospectively retrieved from patient records and presented as the mean (±SD) or the number (percentage), as deemed suitable.

### Ethical Standards

The present study adheres to the ethical principles laid down in the 1964 Declaration of Helsinki and its later amendments. The local ethics committee has granted approval for this study by the Institutional Review Board of the Diagnostic and Treatment Centre for Children and Adults of the Dobrobut Medical Network (Protocol Approval Number: 16 [August 12, 2021]). Patient privacy and confidentiality were protected throughout the study.

### Preoperative Evaluation

In addition to routine preoperative assessments, all patients underwent a computed tomography (CT) scan to screen for atherosclerotic disease and anatomic abnormalities in the aorta and major arterial branches. This step was crucial for planning the CPB strategy and selecting the appropriate peripheral arterial cannulation site, whether via femoral or axillary vessels, and ensuring the safe aortic crossclamping.

**Figure 1:**
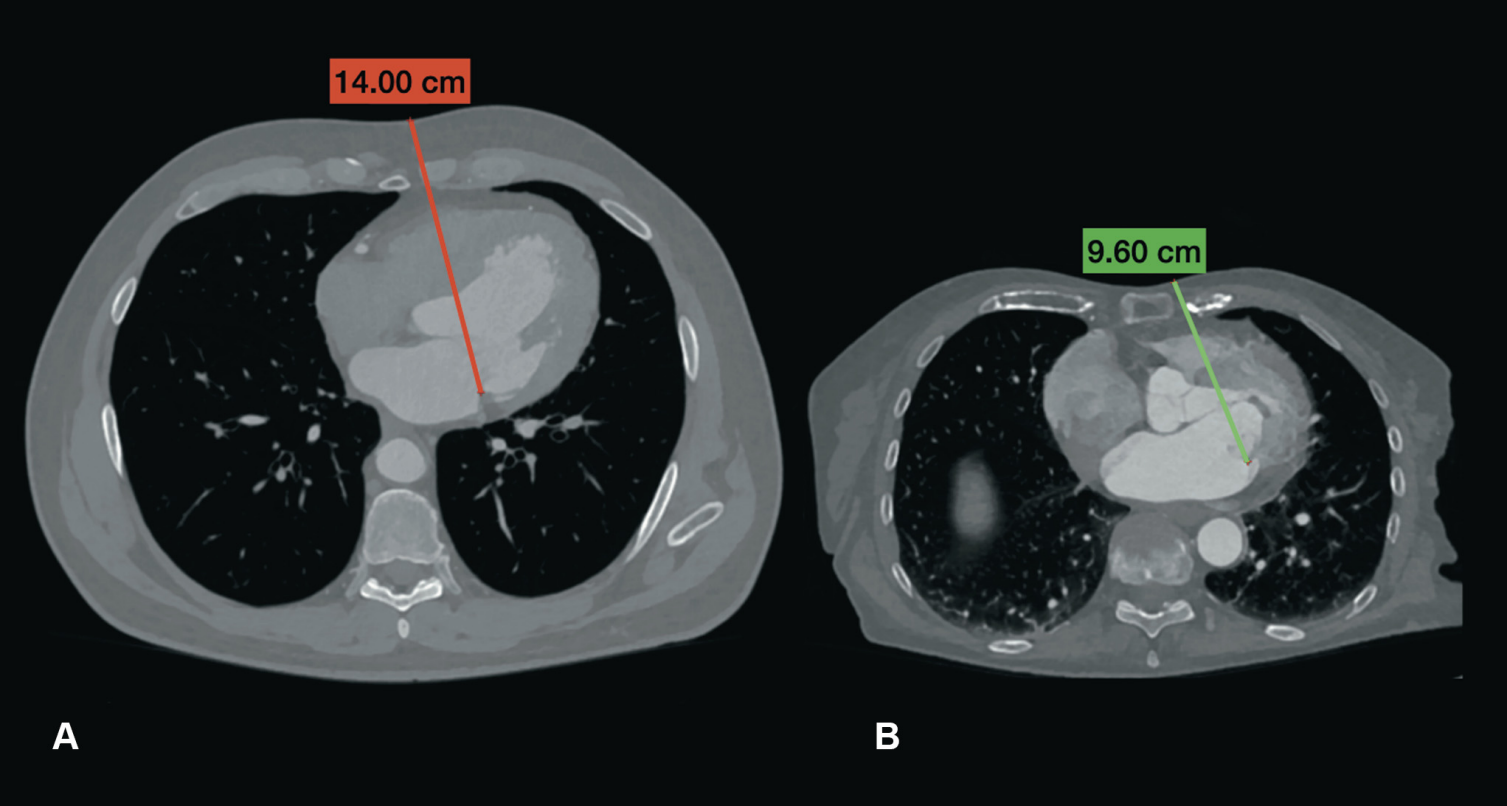
CT distance from the posterior MV annulus to the skin level determines the quality of MV exposure. A, Difficult. B, Excellent (good).

Special attention was dedicated to measuring the distance from the posterior MV ring to the skin level (Figure 1). This measurement was performed to estimate the expected level of MV exposure.

The distance from the skin level to the posterior part of the MV annulus was assessed on transverse CT planes preoperatively. In the initial cases, it was observed that a distance of 14 cm or more was associated with more challenging MV exposure. Subsequently, this parameter was used as a relative cutoff criterion when selecting patients for the LAmT approach for CABG combined with MV surgery.

### Anesthesia

**Figure 2:**
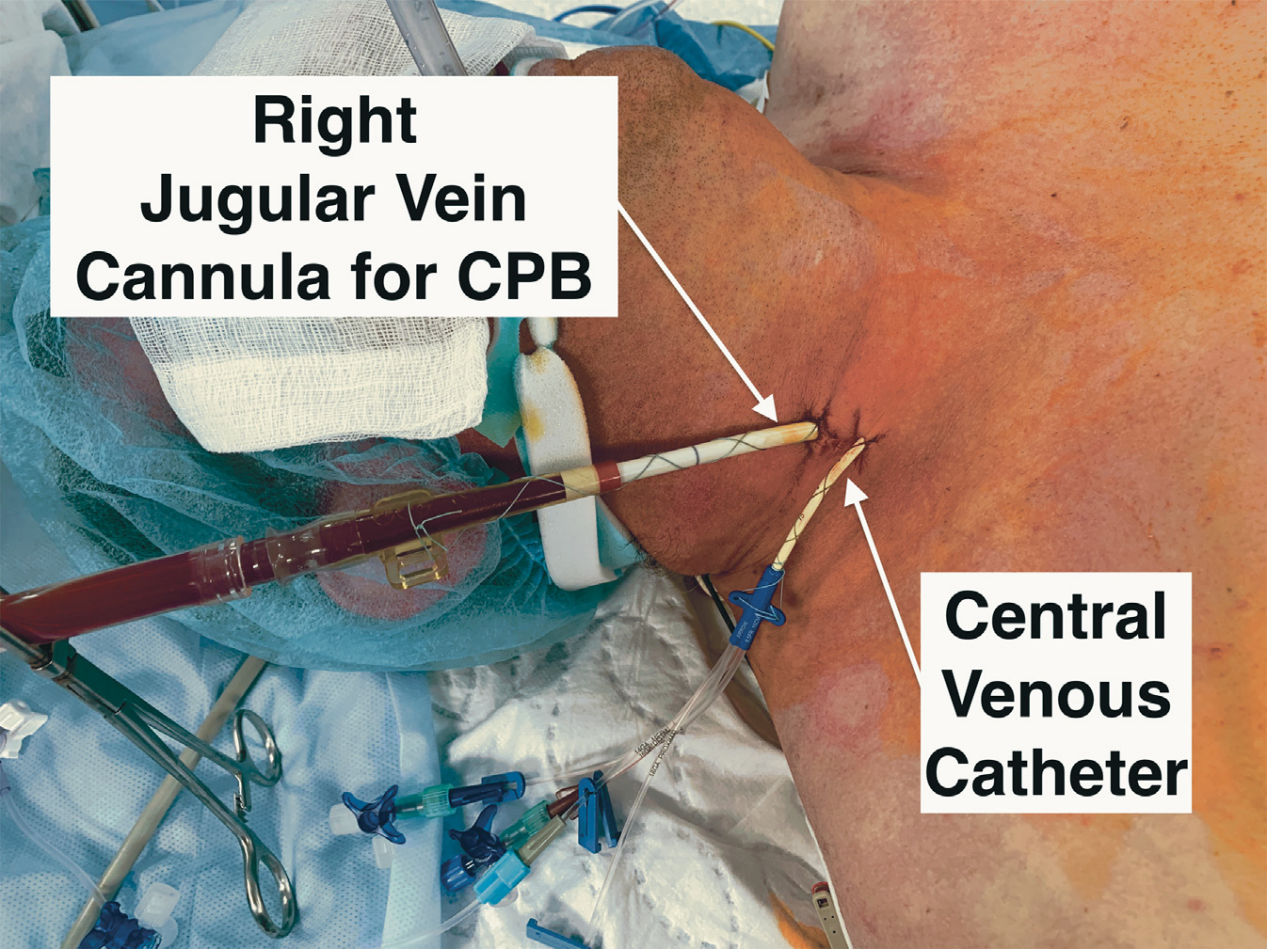
Jugular vein cannula and central venous line position. *CPB*, Cardiopulmonary bypass.

The anesthetic technique involves a low-dose, opioid-based, multimodal approach for both the induction and maintenance of anesthesia. All patients were intubated using a single-lumen endotracheal tube, along with the placement of a 9F Arndt bronchial blocker in the left main bronchus, which is our preferred technique for achieving single-lung ventilation. A jugular vein cannula was preoperatively inserted by an anesthesiologist at the time of central line insertion, and 5000 units of heparin was administered to prevent clotting (Figure 2).

Transesophageal echocardiography was used in all patients. The height of the anterior leaflet of the MV was measured by echocardiography to aid the surgeon in selecting the appropriate mitral ring. The assessment of MV repair was performed intraoperatively by TEE in all patients.

### Surgical Technique

The technique for performing isolated multivessel CABG through the LAmT, known as the “total coronary revascularization via left anterior thoracotomy” (TCRAT-CABG) technique, has been detailed by our team in publications.^3^^,^^4^^,^^6^^,^^7^ In this article, we present a comprehensive description of the technique used for combined procedures including CABG and MV repair or replacement through the LAmT.

All patients were placed in a supine position with an inflatable pad under the left chest. The left arm was positioned at a 90-degree angle to allow for simultaneous radial artery harvesting when necessary.

After administering heparin at a dose of 300 U/kg, CPB lines were inserted. For arterial inflow, an EOPA arterial cannula with a size of 18F to 20F (Medtronic) or a Fem-Flex II Femoral arterial cannula with a size of 16F (Edwards) was used. For venous return, a Bio-Medicus multistage femoral venous cannula with a size of 25F or a Bio-Medicus One-Piece femoral venous cannula with a size of 21F (both from Medtronic) were used.

To access the heart and left internal thoracic artery (LITA) during the TCRAT-CABG procedure, a small skin incision, measuring 7 to 8 cm, was made anteriorly along the 4th intercostal space in men or under the breast in women, commencing from the left sternal edge. The subcutaneous tissue was then mobilized, and the pectoral muscle was split along its fibers. The chest was entered through the fourth intercostal space without rib resection and a rib spreader retractor with 4- to 5-cm wide blades (eg, the Idol, Babliak RETRACTOR or TSI, CT-0100: Pivoting Retractor) was inserted. It is preferred to use a rib spreader with concave retractor blades, because it facilitates surgical exposure and reduces the risk of rib breakage.

LITA harvesting was done with a skeletonized or semiskeletonized technique using a LITA harvesting retractor (Idol Babliak IMA Lifting System with Babliak IMA Upper Retractor Blade and Babliak IMA Lower RETRACTOR Blade or TSI, CT-1705: CT Lift System), long conventional surgical instruments, a diathermy with a 15-cm tip extension and single lung ventilation. CPB was initiated at the end of LITA harvesting to facilitate exposure of the proximal portions of the conduit. Vacuum-assisted venous return was routinely used during CPB.

During the procedure, the pericardium was opened in a T-shaped manner from the apex to the ascending aorta. The ascending aorta was encircled with tape and crossclamped using a transthoracic aortic clamp (Idol, Babliak Aortic Clamp, 80-mm right Curved 2×3 DeBakey jaw, locked, 230-mm working length). This clamp was inserted through the second intercostal space between the midclavicular and anterior axillary lines. Cold blood antegrade intermittent cardioplegia was administered every 15 to 25 minutes or after each distal anastomosis.

The left ventricle was vented through the aortic cardioplegia cannula and separate encircling tapes were placed around the superior vena cava, inferior vena cava, and left pulmonary veins. The exposure of the right and circumflex coronary arteries was facilitated by positioning the tapes under the heart and pulling them to bring the arteries closer to the surgical field. All coronary anastomoses were performed using conventional instruments and techniques. First, all distal anastomoses to the lateral and inferior wall targets were completed before MV repair or replacement, then MV correction was performed, and LITA to left anterior descending artery anastomosis or T-shunt, if needed, were performed after MV surgery. At this point, the surgeon shifted from the left side to the right side of the operating table, and MV correction was executed.

**Figure E1:**
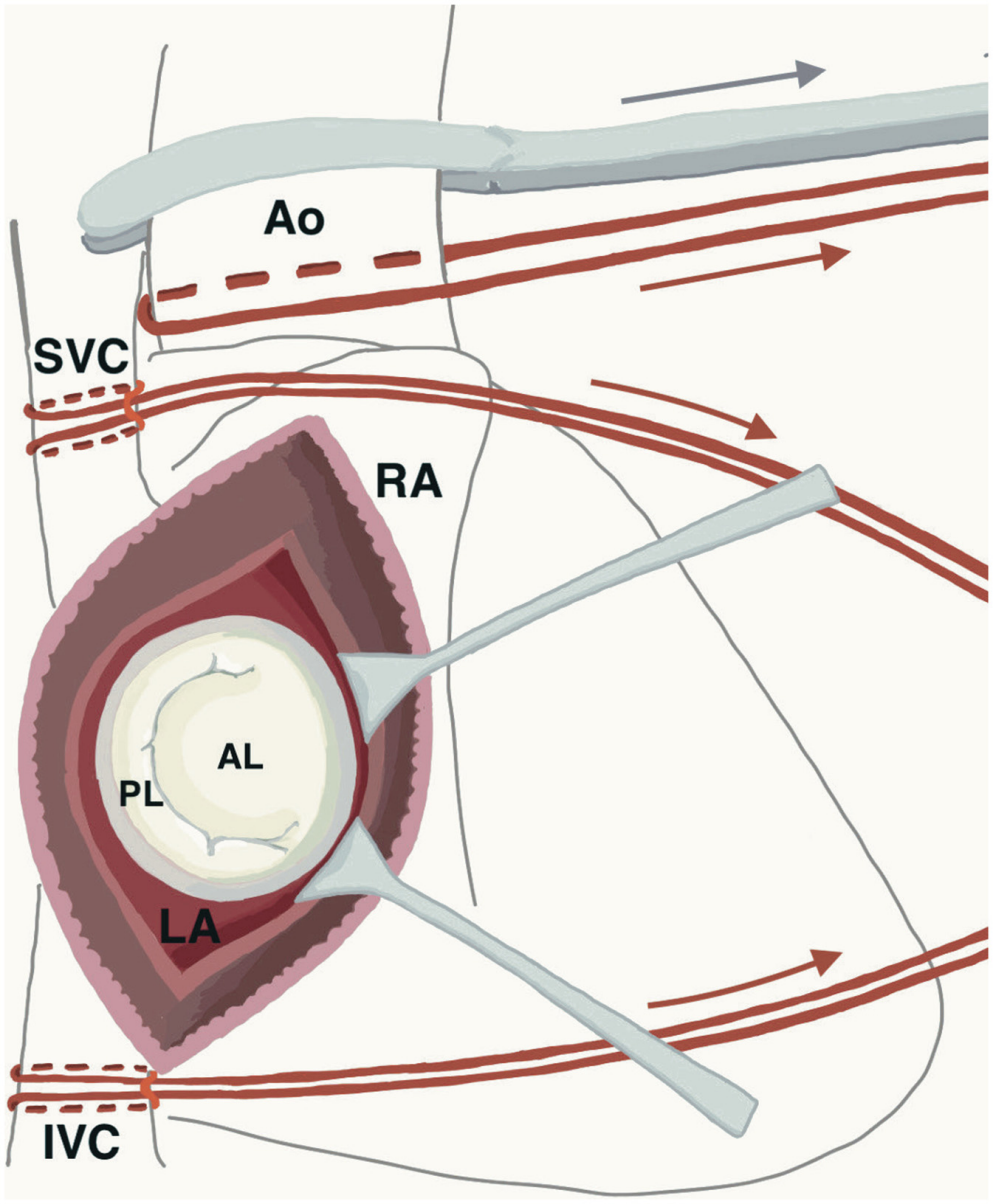
Schematic picture of MV exposure. Tapes are used for displacement of the heart to the left to position the MV in the middle of left anterior thoracotomy. *Ao*, Ascending aorta; *SVC*, superior vena cava; *RA*, right atrium; *AL*, anterior leaflet of the mitral valve; *PL*, posterior leaflet of the mitral valve; *LA*, left atrium; *IVC*, inferior vena cava.

MV access was achieved through the right atrium and interatrial septum. Both the inferior vena cava and superior vena cava tapes were pulled out of the thoracotomy using a tape loop. The tape encircling the ascending aorta was led out through the hole of the transthoracic aortic clamp and pulled to the left to optimize MV exposure (Figure E1). This maneuver shifted the heart to the left chest, moving the right atrium toward the middle of the operative field. Once the right atrium was opened and the interatrial septum was vertically incised, starting from the fossa ovalis, the septum was retracted using 1 or 2 small hooks or stay sutures to expose the MV. Conventional or long-shafted instruments were used for MV repair or replace.

After implanting the ring/semiring or prosthesis and closing the interatrial septum, suture annuloplasty of the tricuspid valve was performed, if planned, followed by closure of all chambers. Chambers closure were more easily performed from the left side of the table, necessitating the surgeon’s return to the left side. Finally, the LITA to left anterior descending artery anastomosis was carried out, and a T-shunt between the LITA and radial was done, if needed, also, always during aortic cross-clamping.

Upon unclamping the aortic, if proximal anastomoses were required, they were performed on the beating heart using a conventional side-biting clamp. The conventional running technique with 6-0 or 7-0 Polypropylene sutures and standard coronary instruments was used. The aortic clamp and tape were pulled to improve the exposure of the aorta.

CPB weaning was performed with double-lung ventilation. Transesophageal echocardiography was performed after the CPB weaning and MV repair or replacement was evaluated. A single drainage tube (preferably a BLAK Silicone Drain with a round hubless design, 24F in size) was inserted through the second intercostal space at the site where the transthoracic aortic clamp had been applied. At the end of the procedure, single lung ventilation was resumed to assist in achieving surgical hemostasis.

## Results

The minimally invasive MV surgery approach through the right atrium and interatrial septum, along with concomitant coronary revascularization via LAmT, was successfully performed in all 24 patients without requiring conversion to sternotomy. Of the 24 patients, 22 (91.7%) underwent MV repair, and the remaining 2 (8.3%) received MV replacement (1 biological and 1 mechanical MV prosthesis). In 10 cases (41%), conventional surgical instruments were used, whereas long-shafted instruments were necessary in the remaining 14 cases (59%), with a knot-pusher being used in 9 cases (37.5%). CT distance from the skin level to MV posterior annulus close to 14 cm was identified as a technical difficulty marker, requiring the use of long-shafted instruments. According to measurements on CT data, in our patient group, this distance was 12.5 ± 1.22 cm (range, 9.95-14.5).

In this series, the prevalence of multivessel disease was notable, with 58% of patients presenting with triple-vessel disease and 33% with double-vessel disease (Table 1). All patients achieved concomitant complete revascularization with a mean number of distal anastomoses of 2.54 ± 0.7 (range, 1-4). Intraoperative parameters are presented in Table 2. The MV repair techniques and coronary conduits used for CABG are presented in Tables 3 and 4.

**Table 1:** Preoperative characteristics

Variable	All patients (N = 24)
Age, y	65.5 ± 9.8 (40; 80)
Female	6 (25%)
Arterial hypertension	16 (66.7%)
BMI >30	10 (42%)
Diabetes mellitus	4 (17%)
Ejection fraction <40%	7 (29.2%)
Severe MV insufficiency	24 (100%)
Triple-vessel disease	14 (58%)
Double-vessel disease	8 (33%)
Atrial fibrillation	7 (29.2%)
Distance from skin level to MV posterior annulus, cm	12.5 ± 1.22 (9.95; 14.5)

Data are presented as mean ± SD (min; max) or n (%). *BMI*, Body mass index; *MV*, mitral valve.

**Table 2:** Intraoperative data

Variable	All patients (N = 24)
Operative time, min	341 ± 41 (285; 420)
CPB time, min	231 ± 38 (172; 316)
Crossclamp time, min	127 ± 23 (80; 169)
Distal anastomoses	2.54 ± 0.7 (1; 4)
Conversion	0

Data are presented as mean ± SD (min; max) or *n* (%). *CPB*, Cardiopulmonary bypass.

**Table 3:** Mitral valve repair techniques in 22 patients

Variable	Patients with MV repair (N = 22)
Ring	10 (45.5)
Semiring	12 (54.5)
Artificial chords	1 (4.5)
Alfieri stitch	2 (9)

Data are presented as n (%).

**Table 4:** Conduits for coronary artery bypass grafting in 24 patients

Variable	All patients (N = 24)
LITA	21 (87.5)
Radial artery	3 (12.5)
Vein	20 (83.3)

Data are presented as n (%). *LITA*, Left internal thoracic artery.

Based on intraoperative TEE assessment, postrepair MV insufficiency was absent in 19 patients (86%) and mild in 3 patients (14%). Coaptation length was more than 7 mm in 4 patients (18.2%), between 5 and 7 mm in 16 patients (72.7%), and less than 5 mm in 2 patients (9.1%). No further interventions were undertaken in all patients.

The mean postoperative intensive care unit and hospital length of stay were 1.87 ± 0.69 days and 6.25 ± 1.86 days, respectively. On average, chest tube drainage was 249.5 ± 87.5 mL for the first 12 hours after surgery. The incidence of pleural effusion and new atrial fibrillation was 17% and 42%, respectively. There was no pericarditis, perioperative stroke, or clinical MI. There were no cases of revision for bleeding and no hospital or 30-day mortality (Table 5).

**Table 5:** Early postoperative outcomes

Variable	All patients (N = 24)
Mechanical ventilation time, h	7.5 ± 5.1 (2; 18)
Chest tube drainage 12 h, mL	249.5 ± 87.5 (110; 470)
ICU length of stay, d	1.87 ± 0.69 (1; 4)
Hospital length of stay, d	6.54 ± 1.86 (4; 10)
New atrial fibrillation	10 (42)
Pericarditis	0
Pleural effusion aspirated	4 (17)
Stroke or transitory ischemic attack	0
Clinical myocardial infarction	0
Mortality	0

Data are presented as mean ± SD (min; max) or n (%). *ICU*, Intensive care unit.

## Discussion

The main advantage of the sternum-sparing approach is to reduce the surgical trauma and to eliminate the risk of osteomyelitis and deep sternal wound infection.^1^ The main finding of this article is that the MV can be surgically accessed through the LAmT approach and that satisfactory MV exposure needed for the implantation of a ring or prosthesis could be obtained. Associated procedures can also be performed through this approach, making combined valve and coronary surgery possible within a single minithoracotomy.

Previous reports about combined MV and CABG surgery with sternum-sparing techniques included different strategies. Bilateral thoracotomies have been described by Smit and colleagues^7^ and Squiccimarro and colleagues.^8^ A combination of percutaneous coronary implantation with robotic MV repair or replacement has been described by Adams and colleagues^9^ or with transapical NeoChords MV repair by Pradegan and colleagues.^10^ Simultaneous MIDCAB procedure and transapical NeoChords have been described by Albertini and colleagues.^11^ Combining coronary beating heart surgical revascularization through the left anterolateral thoracotomy and followed by Tendyne transapical MV replacement therapy has been described by Miazza and colleagues.^12^ The main disadvantage of these strategies is the high selection of patients that has resulted from the high technical complexity of the procedures or surgical and interventional procedural limitations.

The mean aortic crossclamp time of 127 minutes in our study should be taken into consideration. Recently, it was demonstrated that aortic crossclamp time was associated with mortality as well as postoperatively impaired cardiac and renal function in the Mini-Mitral International Registry.^13^ According to this study, the mortality increasing significantly after 120 minutes and implementing measures to reduce crossclamp time may improve outcomes.

Taking into consideration our experience, long crossclamping times in combined valve and coronary cases are unavoidable; thus, focus on the improvement of myocardial protection techniques is equally important. Our preferred method for all cases with long crossclamping times is multi-dose cold blood cardioplegia.

Our aim was to reduce the technical difficulties and high selectivity in using the sternum-sparing approach for combined MV and CABG surgery. As a result, 24 (82.8%) of our patients with combined MV and CABG surgery were operated through a single minithoracotomy and 5 (17.2%) through a sternotomy.

This study includes all combined MV and coronary patients starting from the first one operated through a single left minithoracotomy. Initially, we planned to use this approach only for ischemic mitral insufficiency and mitral ring implantation or replacement.

After the first experience, we understand that MV leaflets, papillary muscles, and chords can all be clearly visualized, and complex repairs in selected patients, including chords implantation, can be performed through the left minithoracotomy. Nevertheless, for isolated MV surgery our preferred approach is a right minithoracotomy.

The CT distance from skin level to MV posterior annulus of more than 14 cm was identified by the surgeon as a need for long-shafted instruments usage for MV repair or replacement, which indirectly indicates the increase in technical difficulties. This should be considered when planning the MV surgery for patients with complex valve pathology and highlights the importance of careful patient selection and preoperative planning to ensure the success of the procedure and reproducibility in general.

Additional factors that may improve or impair the MV exposure are left atrial size and stiffness of chest wall. These factors have not been analyzed in this small, first study. The experience of cardiac surgeon with routine TCRAT-CABG through the left anterior thoracotomy seems to be an important factor to start performing combined procedures through the same incision.

In addition, patients with atherosclerotic ascending aorta and patients who have previously undergone open surgery are not candidates for the technique described in this article.

Regarding the other benefits of this approach except avoiding sternotomy, we may preliminary state that postoperative recovery is similar to other patients who receive a cardiac surgical procedure through the small right of left thoracotomy. Further studies on patient satisfaction are required.

## Conclusions

Overall, our results suggest that this new approach through the LAmT is one of the alternatives to traditional sternotomy for concomitant MV procedures and CABG. However, it is not suitable for all patients, and careful patient selection and surgical expertise are required to ensure the best possible outcomes. Further studies with larger patient cohorts are needed to confirm the reproducibility and efficacy of this approach.

### Webcast

You can watch a Webcast of this AATS meeting presentation by going to: https://www.aats.org/resources/new-approach-for-mitral-valve-repair-replacement-through-the-left-anterior-minithoracotomy-and-transseptal-right-atrial-incision.

**Figure:**
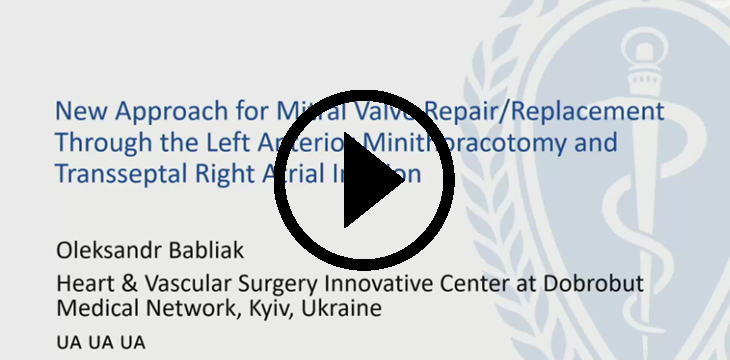

## Conflict of Interest Statement

Dr Babliak provided consultation services to Idol Company while being involved in the development and improvement of surgical tools used in TCRAT-CABG surgery. All other authors reported no conflicts of interest.

The *Journal* policy requires editors and reviewers to disclose conflicts of interest and to decline handling or reviewing manuscripts for which they may have a conflict of interest. The editors and reviewers of this article have no conflicts of interest.

## References

[1] Walther T., Falk V., Mohr F.W. (2006). Minimally invasive surgery for valve disease. Curr Probl Cardiol.

[2] Babliak D., Babliak O., Demianenko V., Marchenko A. (2023). Coronary artery bypass grafting and mitral valve replacement via a left anterior minithoracotomy. Multimed Man Cardiothorac Surg.

[3] Babliak O., Demianenko V., Melnyk Y., Revenko K., Babliak D., Stohov O. (2020). Multivessel arterial revascularization via left anterior thoracotomy. Semin Thorac Cardiovasc Surg.

[4] Babliak O., Demianenko V., Melnyk Y., Revenko K., Pidgayna L., Stohov O. (2019). Complete coronary revascularization via left anterior thoracotomy. Innovations.

[5] Carpentier A. (1983). Cardiac valve surgery–the “French correction.”. J Thorac Cardiovasc Surg.

[6] Babliak O., Demianenko V., Melnyk Y., Revenko K., Pidgayna L., Stohov O. (2019). Total coronary revascularization via left anterior thoracotomy: practical aspects. Multimed Man Cardiothorac Surg.

[7] Smit P.J., Shariff M.A., Nabagiez J.P., Khan M.A., Sadel S.M., McGinn J.T. (2013). Experience with a minimally invasive approach to combined valve surgery and coronary artery bypass grafting through bilateral thoracotomies. Heart Surg Forum.

[8] Squiccimarro E., Margari V., Paparella D. (2022). Bilateral mini-thoracotomy for combined minimally invasive direct coronary artery bypass and mitral valve repair. Eur J Cardiothorac Surg.

[9] Adams C., McClure R.S., Goela A., Bainbridge D., Kostuk W.J., Kiaii B. (2010). Simultaneous robotic-assisted mitral valve repair and percutaneous coronary intervention. Innovations (Phila).

[10] Pradegan N., D'Onofrio A., Longinotti L., Evangelista G., Mastro F., Fiocco A. (2021). Feasibility of percutaneous coronary intervention before mitral NeoChord implantation: single-center early results. J Card Surg.

[11] Albertini A., Amoncelli E., Piccinini L., Caprili L. (2018). Combined off-pump minimally invasive coronary artery bypass grafting and mitral valve repair with NeoChord via a left anterolateral small thoracotomy. Interact Cardiovasc Thorac Surg.

[12] Miazza J., Koechlin L., Jeger R.V., Reuthebuch O.T. (2022). First-in-man concomitant mitral valve replacement and coronary artery bypass grafting using a single minimally invasive access. Eur J Cardiothorac Surg.

[13] Doenst T., Berretta P., Bonaros N., Savini C., Pitsis A., Wilbring M. (2023). Aortic cross-clamp time correlates with mortality in the Mini-Mitral International Registry. Eur J Cardiothorac Surg.

